# Optimizing the PMMA Electron-Blocking Layer of Quantum Dot Light-Emitting Diodes

**DOI:** 10.3390/nano11082014

**Published:** 2021-08-06

**Authors:** Mariya Zvaigzne, Alexei Alexandrov, Anastasia Tkach, Dmitriy Lypenko, Igor Nabiev, Pavel Samokhvalov

**Affiliations:** 1 Laboratory of Nano-Bioengineering, Institute of Engineering Physics for Biomedicine, National Research Nuclear University MEPhI (Moscow Engineering Physics Institute), 31 Kashirskoe Highway, 115409 Moscow, Russia; taa025@campus.mephi.ru (A.T.); igor.nabiev@univ-reims.fr (I.N.); 2 Laboratory of Electronic and Photonic Processes in Polymeric Nanomaterials, A.N. Frumkin Institute of Physical Chemistry and Electrochemistry of the Russian Academy of Sciences, 31, bld.4, Leninsky Prospect, 119071 Moscow, Russia; klays007@gmail.com (A.A.); lypenko@phyche.ac.ru (D.L.); 3 Laboratory of Immunopathology, I.M. Sechenov First Moscow State Medical University, 8-2 Trubetskaya Str., 119991 Moscow, Russia; 4 Laboratoire de Recherche en Nanosciences, Université de Reims Champagne-Ardenne, 51 rue Cognacq Jay, 51100 Reims, France

**Keywords:** quantum dots, QDLED, electron-blocking layer, PMMA

## Abstract

Quantum dots (QDs) are promising candidates for producing bright, color-pure, cost-efficient, and long-lasting QD-based light-emitting diodes (QDLEDs). However, one of the significant problems in achieving high efficiency of QDLEDs is the imbalance between the rates of charge-carrier injection into the emissive QD layer and their transport through the device components. Here we investigated the effect of the parameters of the deposition of a poly (methyl methacrylate) (PMMA) electron-blocking layer (EBL), such as PMMA solution concentration, on the characteristics of EBL-enhanced QDLEDs. A series of devices was fabricated with the PMMA layer formed from acetone solutions with concentrations ranging from 0.05 to 1.2 mg/mL. The addition of the PMMA layer allowed for an increase of the maximum luminance of QDLED by a factor of four compared to the control device without EBL, that is, to 18,671 cd/m^2^, with the current efficiency increased by an order of magnitude and the turn-on voltage decreased by ~1 V. At the same time, we have demonstrated that each particular QDLED characteristic has a maximum at a specific PMMA layer thickness; therefore, variation of the EBL deposition conditions could serve as an additional parameter space when other QDLED optimization approaches are being developed or implied in future solid-state lighting and display devices.

## 1. Introduction

Fluorescent semiconductor nanocrystals (NCs) or quantum dots (QDs) have plenty of advantageous properties, such as the possibility of tuning the luminescence wavelength by varying the physical size of the NCs and the capacity for forming stable colloidal solutions, which makes it possible to obtain coatings by inexpensive solution-process methods, and make QDs promising materials in optoelectronic, bioimaging, lighting, and other applications [1,2,3,4,5]. Today, light-emitting devices (LEDs) based on organic compounds (OLEDs) prevail in commercial lighting and display appliances. Quantum dots outperform traditional organic dyes in terms of the width of the absorption spectrum, molar extinction, and photostability. Thus, quantum dots are expected to be promising candidates to overcome the material stability issues typical of OLEDs, such as drastic efficiency roll-off at high current densities and mediocre operational lifetimes. Moreover, due to their inorganic nature, QDs are much more thermally stable materials, which makes it possible to increase the brightness of QD-based LEDs by increasing the current density in the device. In addition, QDs have quite narrow fluorescence and electroluminescence spectra and, hence, are promising components of displays or illuminators with a wide color gamut. To fully exploit the superior properties of QDs, a number of QD-based LED (QDLED) structures with different device and material configurations have been developed [6]. A typical QDLED-emitting layer represents a thin QD film sandwiched between two charge-transport layers, and its interaction with them may cause luminescence quenching through various nonradiative pathways, such as the Auger recombination [7,8], QD charging [7], and charge and/or energy transfer from QDs to the charge-transport materials [7,9], and so forth.

One of the main shortcomings of QD-based LEDs is imbalance between the rates of charge carrier injection and transport [7], which leads to the formation of excess charges (electrons or holes) in the emitting QD layer and quenching of QD radiation due to the aforementioned nonradiative processes. This phenomenon leads to a significant decrease in radiation efficiency, especially at high current densities, and, hence, to overall low performance of the QDLEDs. In most modern QDLED configurations [10], this imbalance mainly results from a larger potential barrier for the injection of holes into the QD layer than for the injection of electrons, as well as a higher mobility of electrons in the electron transport layer (ETL), usually based on ZnO, compared to the hole mobility in organic hole transport layers (HTL) [11]. One of the approaches to solving this problem is the introduction of an electron-blocking layer (EBL). The EBL materials and methods of its integration into the QDLED structure vary widely. For example, it has been shown that the addition of a 4,4,4-tris(N-carbazolyl)-triphenylamine (TcTa) EBL between the hole-transporting layer and the light-emitting QD layer [12] or a combined hole-transporting and electron-blocking layer of deoxyribonucleic acid (DNA) complexed with cetyltrimetylammonium (CTMA) [13] enhances efficiency due to the reduction of electron overflow and improvement of hole injection. Another approach is to insert an EBL between the ETL and the QD layer to restrict the flow of electrons. In the framework of this approach, Al_2_O_3_ [14] and poly(methyl methacrylate) (PMMA) [15] have been demonstrated to be effective materials for the EBL. However, a thin film of poly (methyl methacrylate) (PMMA) is more often used as an EBL [15,16], because the positions of its energy levels provide a high potential barrier for electron injection into the emitting layer for most types of QDs. In addition, PMMA is soluble, such as in acetone, which makes it possible to apply PMMA onto the underlying QD layer without partially dissolving or deforming the latter.

The first introduction of a PMMA electron blocking layer into the QDLED structure was reported by Dai et al. [15], who fabricated QDLEDs with a 6 nm PMMA insulating layer between a CdSe/CdS core/shell QD layer and a ZnO ETL to optimize the charge balance in the device. They compared the hole mobility in an HTL consisting of poly-TPD (1·10^−4^ cm^2^·V^−1^·s^−1^) and PVK (2.5·10^−6^ cm^2^·V^−1^·s^−1^) with the electron mobility in an ETL based on a ZnO nanocrystal film (∼1.8·10^−3^ cm^2^·V^−1^·s^−1^), and concluded that the insertion of the PMMA layer may lead to excess electron injection into the QD emissive layer. To confirm this assumption, they measured and compared the current densities of the electron-only devices (ITO/Al/QDs/ZnO/Al) and hole-only devices (ITO/PEDOT:PSS/poly-TPD/PVK/QDs/Pd). In this case, the addition of the PMMA layer between the ETL and the QD layer to optimize charge balance did not cause any considerable changes in either the turn-on voltage or the brightness in comparison with the control QDLEDs without the PMMA layer. However, for equally bright QDLEDs, the current density in the control device was much greater, which indicated that the efficiency of this device was substantially lowered by the excess electron current. Thus, the efficiency of the EBL-based approach in terms of enhancing the performance of QDLEDs was confirmed. In addition, the stability of the devices without the PMMA layers was relatively poor, and it was improved 20-fold by adding the PMMA EBL.

Rahmati et al. [16] presented a new QDLED architecture with multiple PMMA EBLs sandwiched between a pair or more of QD layers. The authors developed QDLED structures with one, two, and three PMMA layers and showed that a device containing two PMMA and three QD layers had the best current efficiency of 17.8 cd·A^−1^ and a luminance of 194,038 cd·m^−2^. The substantial improvement of QDLED performance was mainly attributed to the addition of the PMMA EBL, which reduced the backward electron leakage from the active QD region and enhanced electron confinement, leading to an increased electron concentration in the QD active layers and a higher radiative recombination rate. It is worth noting that the aforementioned QDLED configuration where the EBL is sandwiched between a pair of QD EMLs could be employed in the design of white QDLEDs by combining isolated blue/green/red QD layers separated by two PMMA spacers into a complex emissive layer [16].

Although it has been demonstrated that adding a PMMA layer to the QDLED structure may significantly improve the performance of QDLEDs, this approach needs further optimization and detailed study to rationally exploit the PMMA electron-blocking capacity. Here, we studied the correlations between the parameters of the PMMA EBL deposition, such as the PMMA solution concentration, and the most important performance characteristics of QDLEDs.

## 2. Materials and Methods

### 2.1. Synthesis of CdSe/ZnS/CdS/ZnS (CdSe/MS) QDs

The synthesis of CdSe cores with a diameter of 2.3 nm was carried out by the hot injection technique using cadmium hexadecylphosphonate and trioctylphosphine as precursors at a temperature of 240 °C; the procedure is described in more detail in [17]. After the synthesis, the separation and purification of the CdSe cores were carried out by means of reprecipitation of nanocrystals and subsequent gel permeation chromatography, after which their surface was treated with oleylamine in the presence of sodium borohydride to replace the hexadecylphosphonic acid residues with oleylamine, which facilitated further growth of inorganic shells. Then, after additional purification steps, the CdSe cores were placed into a reaction mixture of 1-octadecene and oleylamine (1:1, *v*/*v*) for growing the shells. After accurate quantification of CdSe cores in the reaction solution using the approach described in [18], we calculated the quantities of precursors required for obtaining QDs with the desired shell structure. The shells were grown at 170 °C in an argon atmosphere at an average growth rate of 1 monolayer per 30 min. After the synthesis and isolation of QDs from the crude solution, the organic ligands were replaced with hexadecylammonium palmitate (HDA-PA), which reduced the sensitivity of the optical properties of QDs to atmospheric exposure during long-term storage. The luminescence and absorption spectra, as well as the transmission electron microscopy (TEM) image of the obtained QDs are shown in Figures S1 and S2, provided in the Supporting Information (SI) file.

### 2.2. Fabrication of QDLED Devices

Glass substrates with an indium tin oxide (ITO) layer were preliminarily cleaned by treatment in an ultrasonic bath and then in oxygen plasma. Then, a hole-injecting layer of PEDOT:PSS (poly(3,4-ethylenedioxythiophene): poly(styrene sulfonate)) was deposited on the substrates by spin-coating at 2000 rpm, followed by annealing at 110 °C for 10 min. The film thickness was 40 nm. The substrates coated with a PEDOT: PSS layer were transferred into a glove box containing argon (O_2_ < 1 ppm, H_2_O < 1 ppm). Next, hole transport layers of poly-TPD (poly(N,N′-bis-4-butylphenyl-N,N′-bisphenyl) benzidine, solution in chlorobenzene, 8 mg/mL) and PVK (poly (vinylcarbazole), solution in o-xylene, 1.5 mg/mL) were deposited alternately by spin-coating at 2000 rpm. Layers of poly-TPD (30 nm) and PVK (5 nm) were annealed at 100 °C for 10 min before applying the next layer. Then, a QD layer was applied from a solution in n-octane (20 mg/mL) by spin-coating at 2500 rpm and annealed at 100 °C for 10 min. The QD film thickness was 40 nm. The following PMMA (Sigma Aldrich, Saint Louis, MO, USA, average M_w_ ~ 120,000 Da) layer was applied by spin-coating from a solution in acetone at 3500 rpm and then annealed at 100 °C for 10 min. The concentration of the acetone solution of PMMA varied from 1.2 mg/mL to 0.05 mg/mL to obtain different blocking-layer thicknesses. A 50 nm electron-transport layer (ETL) was applied from solutions of ZnO nanoparticles in isopropyl alcohol (25 mg/mL) by spin-coating at 1000 rpm followed by annealing at 60 °C for 10 min. Finally, an aluminum cathode with a thickness of 80 nm was deposited onto the ETL through a shadow mask by thermal evaporation in vacuum (2·10^−6^ mbar).

### 2.3. Instrumental Methods

The luminescence spectra were measured using an Agilent Cary Eclipse spectrofluorimeter. The absorption spectra were measured using an Agilent Cary 60 UV-Vis spectrophotometer. Transmission electron microscopy (TEM) images were obtained on a JEOL JEM-2100F (JEOL Ltd., Tokyo, Japan) instrument operated at 200 kV acceleration voltage. TEM specimens were prepared by drop-casting a solution of QDs in hexane onto carbon/Formvar-coated 200 mesh copper TEM grids. The voltage–current and voltage–brightness characteristics were measured with a Keithley 2601 SourceMeter 2601 (Keithley Instruments, Inc., Solon, OH, USA), a Keithley 485 picoampermeter (Keithley Instruments, Inc., Solon, OH, USA), and a TKA-04/3 luxmeter–brightness meter (Scientific Instruments “TKA”, St. Petersburg, Russia). The preparation of QDLED samples and measurements of their characteristics were performed at room temperature in an argon atmosphere. The film thicknesses were determined by ellipsometry using an MII-4 interferometer (“LOMO”, St. Petersburg, Russia) and by means of a MultiMode V (Bruker Corporation, Billerica, MA, USA) atomic force microscope.

## 3. Results and Discussion

Deposition of an ultimately thin PMMA charge-blocking layer on top or within the emissive QD layer by means of solution processes requires the minimum possible distortion of the QD layer to achieve efficient performance of the QDLED. Thus, selection of the appropriate solvent for PMMA is of utmost importance. PMMA can be dissolved in a number of organic nonpolar (toluene, chloroform, etc.) and weakly polar (acetone) solvents. In most QDLED configurations, QDs capped with long-chain aliphatic ligands are deposited from nonpolar solvents, such as octane and toluene. Therefore, acetone becomes the solvent of choice for deposition of PMMA because, being polar, it cannot dissolve the underlying QDs and, at the same time, being only mildly polar, it cannot cause severe wrapping or cracking of the underlying thin film of QDs. On the other hand, acetone is quite volatile and has a low viscosity; therefore, it is hard to control the parameters of its deposition by spin-coating other than the concentration of PMMA in solution.

**Figure 1:**
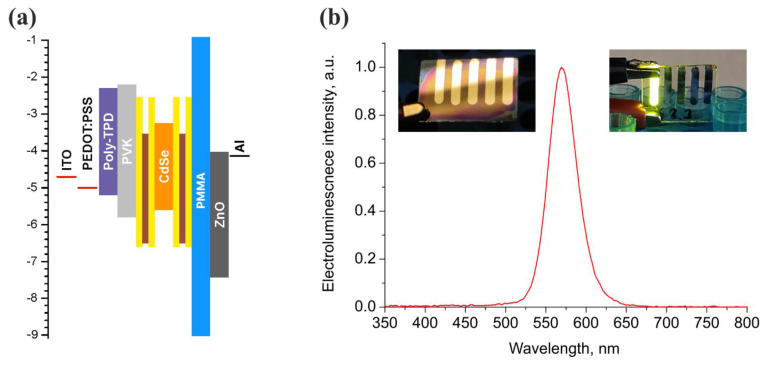
Flat-band energy level diagram of the fabricated QDLEDs (**a**) and electroluminescence spectrum of QDLED with the PMMA electron-blocking layer deposited from a solution with a PMMA concentration of 0.2 mg/mL (**b**). The insets in panel (**b**) show photographs of the device under ambient light (left) and operated at 6 V (right).

The structure of the fabricated QDLED devices is illustrated in Figure 1 along with the schematic of the flat-band energy level diagram of the layers in the device.

We used QDs with the core/multishell structure to eliminate the negative effects on QD fluorescence caused by Auger recombination and surface-trapping [19,20,21], and because our previous studies revealed that this type of QD possessed the optimal characteristics of photostability due to the suppression of charge transfer [4]. In order to increase the efficiency of hole injection into the QD-emitting layer, we added a poly-TPD/PVK bilayer-structured hole-injection layer between QDs and PEDOT:PSS. This configuration creates a gradual step transition between the hole energy levels of QDs and PEDOT:PSS hole-transport layer. A thin layer of ZnO nanoparticles was deposited as an electron transport layer (ETL) because ZnO proved to be the most favorable ETL material due to its high transparency, low work function, and high electron mobility [6,10,15]. To investigate the effect of the PMMA layer preparation routine on the QDLED performance, a series of devices were fabricated. To do this, we varied the concentration of the PMMA solution in acetone from 0.05 to 1.2 mg/mL. Unfortunately, we were unable to measure the exact thickness of the PMMA EBL using the available AFM instrumentation, because the measurement error was higher than the measured value. Otherwise, we may roughly estimate the thickness range of PMMA EBL in our devices as 0.13–3 nm, corresponding to the lower and upper limits of the solution concentrations, respectively. The details of this estimation are given in the SI, the results of the calculation are given in the Table S1. Yet, in the following sections, we prefer to stick to the known experimental concentration values rather than our rough thickness estimations.

**Figure 2:**
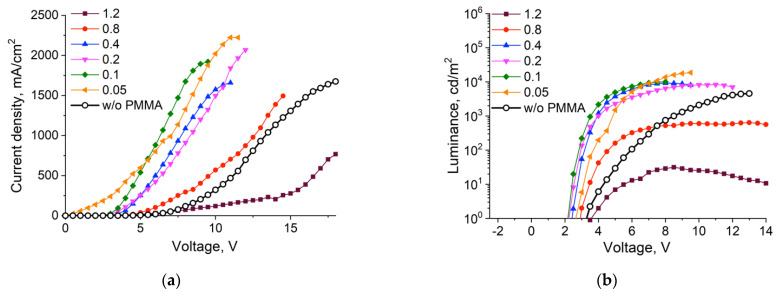
Current density (**a**) and luminance (**b**) versus voltage characteristics of QDLED samples employing a PMMA EBL deposited from PMMA solutions in acetone with different concentrations.

Figure 2a shows the current density–voltage and luminance–voltage characteristics of the devices under investigation. As can be seen, the addition of a PMMA blocking layer in most cases led to an accelerated rise and an overall increase in the current density at all voltages relative to the structure without a blocking layer. Only when we applied the EBL of PMMA from a solution with the highest concentration (1.2 mg/mL) did we observe a drop in this characteristic. These results might be counterintuitive at first glance, because blocking of electron flux through the device by a potential barrier created by the PMMA layer led to an overall increase in the current flow through the whole device. However, previous studies showed that the imbalance between the electron and hole currents led to the accumulation of excess electrons inside the device and interfacial charging [7,11], which, in turn, acts as a counter-driving force for electron currents and leads to less efficient electron transport and injection. EBL, in this case, diminishes the charge flow imbalance and allows the device to operate in the optimal regime, since the PMMA interlayer provides quite a high energy barrier of around 3 eV against electron flow from the ETL (Figure 1). Thus, PMMA EBL can block excess electron flow from ETL to the QD light-emitting layer by reducing the electron current density, which leads to the improved charge carrier balance inside the emissive QD layer, as it was shown for a number of other EBL materials [22,23,24,25].

As can be seen from our data, this optimal charge carrier balance was achieved when EBL was deposited from a solution with a PMMA concentration in the range of 0.1–0.4 mg/mL. In this range, higher PMMA concentrations yield devices with a lower current density but similar luminance. On the other hand, when the PMMA concentration in deposition solution was lowered to 0.05 mg/mL, we observed current leakage even at low voltages (0–2.5 V), which suggested a short circuit due to disturbance of the emissive layer during EBL deposition.

A similar trend was observed for the luminance–voltage characteristics (Figure 2b). In this case, the luminance saturation plateau was reached faster, and the brightness values were higher in QDLED structures fabricated with a blocking PMMA layer. As an exception, QDLED samples employing a PMMA EBL deposited from solutions with concentrations of 0.8 and 1.2 mg/mL exhibited only minor, if any, improvement of this characteristic. In the case of a 0.8 mg/mL solution, sharper growth was observed, but the brightness value did not exceed that for the device without an EBL. These effects may arise from hindered injection of electrons into the emitting layer due to an increase in the thickness of the potential barrier and, as a consequence, a decrease in the probability of carrier tunneling.

**Figure 3:**
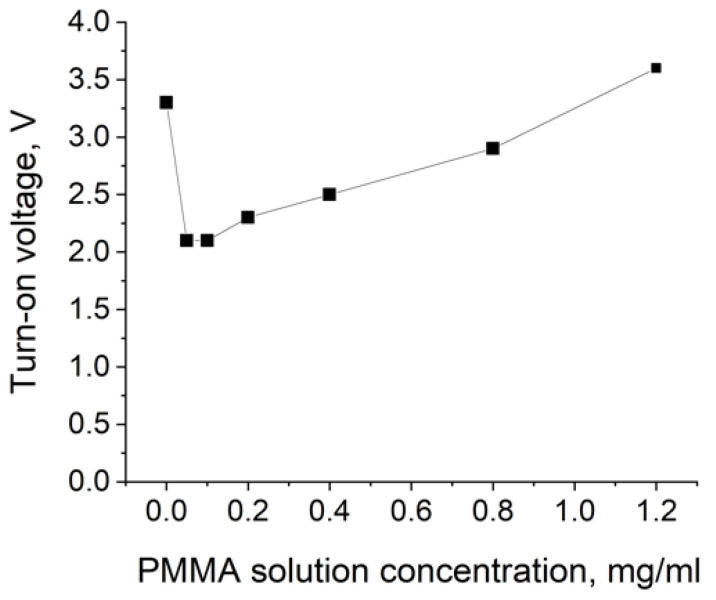
Effect of PMMA solution concentration on the turn-on voltage value of the QDLED device.

The performance parameters of all fabricated QDLED devices are summarized in Table 1. The lowest turn-on voltage of 2.1 V was observed for the two lowest PMMA solution concentrations, 0.1 and 0.05 mg/mL. At the same time, the QDLED structure without a PMMA layer had one of the highest turn-on voltage values, 3.3 V. In general, a distinct minimum was observed in the plot of the turn-on voltage versus the PMMA solution concentration (Figure 3).

**Figure 4:**
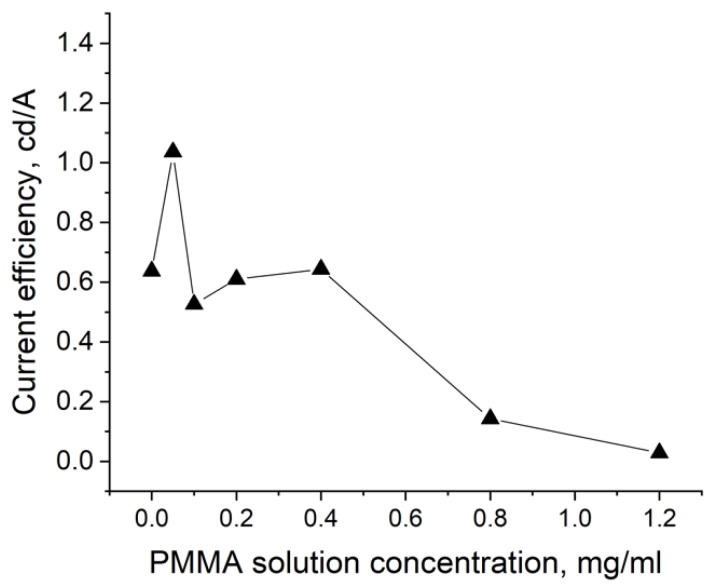
Effect of the PMMA solution concentration on the current efficiency of the QDLED device at 9 V.

**Figure 5:**
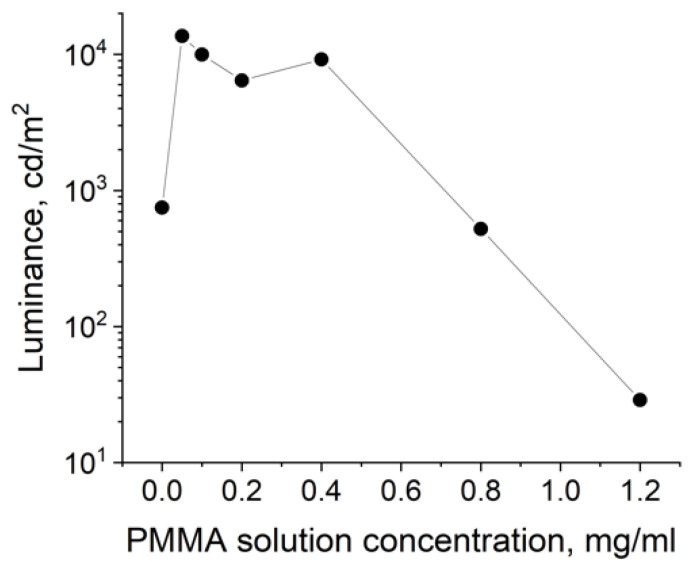
Effect of the PMMA solution concentration on the luminance of the QDLED device at 9 V.

In terms of the maximum current efficiency, the QDLED with an EBL deposited from a 0.4 mg/mL PMMA solution turned out to be the optimal one (Table 1). An increase in PMMA concentration led to a sharp drop of the current efficiency, while its decrease also resulted in a 1.5-fold lower current efficiency. For concentrations of 0.2 and 0.1 mg/mL, there were no significant differences in either current efficiency or turn-on voltage. However, the brightness steadily increased with decreasing PMMA concentration in the EBL deposition procedure. Thus, the maximum brightness in our experiment was 18 671 cd/m^2^, obtained in the case of QDLEDs with an EBL fabricated using a 0.05 mg/mL PMMA solution. This luminance value was four times higher than that for devices without a blocking layer. Figure 4 and Figure 5 show the dependences of the current efficiency and luminance at 9 V on the PMMA solution concentration.

As can be seen, in the case of current efficiency, the apparent maximum is observed for the device where the EBL had the minimum thickness, when it was deposited from a solution with a PMMA concentration of 0.05 mg/mL, and for the device without EBL. Additionally, a local maximum was observed for the device whose EBL was formed from a 0.4 mg/mL PMMA solution. This result suggests that either excess electron injection or over-blocking of the electron current deteriorates charge balance in the QDLEDs and thereby degrades current efficiency values.

Regarding the luminance (Figure 5), addition of even the thinnest PMMA layer to the QDLED led to a drastic increase in this characteristic, apparently due to reducing the probability of the formation of excess charges in the QD emissive layer and preventing the luminescence quenching via nonradiative processes. However, further increase in the concentration of PMMA in the EBL deposition solution resulted in deterioration of this characteristic. This may have been due to the hindered injection of electrons into the emitting layer as a result of an increased thickness of the potential barrier and, as a consequence, a decreased probability of carrier tunneling.

Our findings show that the most important characteristics of QDLEDs can be substantially improved by careful adjustment of the PMMA EBL deposition parameters, such as PMMA solution concentration. Notably, among the QDLEDs studied here, there was no obvious best device in terms of the turn-on voltage, current efficiency, and luminance. Therefore, the addition of the PMMA as an EBL alone should not be considered as a single treatment to improve all the QDLED characteristics, but it may be quite effective if applied along with other optimization approaches. In this case, the PMMA layer deposition parameters should be adjusted according to the requirements of each specific QDLED structure. Our results may be helpful as guidance for the preparation of a PMMA EBL in order to adjust specific QDLED parameters.

## 4. Conclusions

An electron-blocking layer of poly(methyl methacrylate) was added to the standard QDLED structure in order to improve the brightness characteristics and current efficiency. It has been shown that the concentration of the PMMA solution during layer deposition plays a significant role in achieving high QDLED efficiency. Specifically, at a concentration as high as 1.2 mg/mL, the characteristics of the current efficiency and brightness of the QDLEDs dropped significantly relative to a similar device without an EBL. This may be due to the hindered injection of electrons into the emitting layer due to an increase in the thickness of the potential barrier and, as a consequence, a decrease in the probability of carrier-tunneling.

At the same time, a low concentration of the initial PMMA solution leads to a sharp improvement of the characteristics of the QDLEDs, both in terms of brightness and current efficiency and in terms of lowering the turn-on voltage. In terms of current efficiency, the QDLED sample with an EBL deposited from a 0.4 mg/mL PMMA solution turned out to be the optimal one. Apparently, this was why the resultant EBL provided a better balance of the inflow of charge carriers into the QD layer. In the case of the minimum concentration of the PMMA solution, the brightness of the LEDs produced was 18,671 cd/m^2^, which is four times higher than these values for devices without a blocking layer due to reducing the number of charged QDs and probability of nonradiative processes.

## Figures and Tables

**Table 1:** Summary of the performance parameters of the fabricated QDLED devices with and without a PMMA layer deposited from PMMA solutions with different concentrations.

PMMA Solution Concentration, mg/mL	1.2	0.8	0.4	0.2	0.1	0.05	w/o PMMA
Turn-on voltage, V	3.6	2.9	2.5	2.3	2.1	2.1	3.3
Maximum current efficiency, cd/A	0.04	0.18	0.95	0.63	0.73	0.99	0.49
Luminance, cd/m^2^	33	635	9093	8146	9969	18,671	4472

## Data Availability

The data presented in this study are available on request from the corresponding authors.

## Supplementary Materials

The following are available online at https://www.mdpi.com/article/10.3390/nano11082014/s1. Figure S1: Luminescence and absorption spectra of the synthesized CdSe/ZnS/CdS/ZnS QDs; Figure S2: TEM image of the synthesized CdSe/ZnS/CdS/ZnS QDs; Formulas regarding the estimation of the thickness of PMMA electron blocking layers; Table S1: Estimated PMMA EBL layer thickness deposited from PMMA solutions in acetone with different concentrations.
