# Supplementary material for: Optimizing the PMMA Electron-Blocking Layer of Quantum Dot Light-Emitting Diodes
Source: Nanomaterials (Basel). 2021 Aug 6;11(8):2014. doi: 10.3390/nano11082014 (PMC8401809; doi:10.3390/nano11082014)
Supplement: Supplementary file 1 [file nanomaterials-11-02014-s001.zip › nanomaterials-1317252-supplementary.pdf]

nanomaterials

MDPI

Supplementary Materials

Optimizing the PMMA Electron-Blocking Layer of Quantum Dot Light-Emitting Diodes

Mariya Zvaigzne [1,*], Alexei Alexandrov [2], Anastasia Tkach [1], Dmitriy Lypenko [2], Igor Nabiev [1,3,4] and Pavel Samokhvalov [1,*]

1 Laboratory of Nano-Bioengineering, Institute of Engineering Physics for Biomedicine, National Research Nuclear University MEPhI (Moscow Engineering Physics Institute), 31 Kashirskoe Highway, 115409 Moscow, Russia; taa025@campus.mephi.ru (A.T.); igor.nabiev@univ-reims.fr (I.N.)
2 Laboratory of Electronic and Photonic Processes in Polymeric Nanomaterials, A.N. Frumkin Institute of Physical Chemistry and Electrochemistry of the Russian Academy of Sciences, 31, bld.4, Leninsky Prospect, 119071 Moscow, Russia; klays007@gmail.com (A.A.); lypenko@phyche.ac.ru (D.L.)
3 Laboratory of immunopathology, I.M. Sechenov First Moscow State Medical University, 8-2 Trubetskaya Str., 119991 Moscow, Russia
4 Laboratoire de Recherche en Nanosciences, Université de Reims Champagne-Ardenne, 51 rue Cognacq Jay, 51100 Reims, France
* Correspondence: mazvajgzne@mephi.ru (M.Z.); pssamokhvalov@mephi.ru (P.S.)

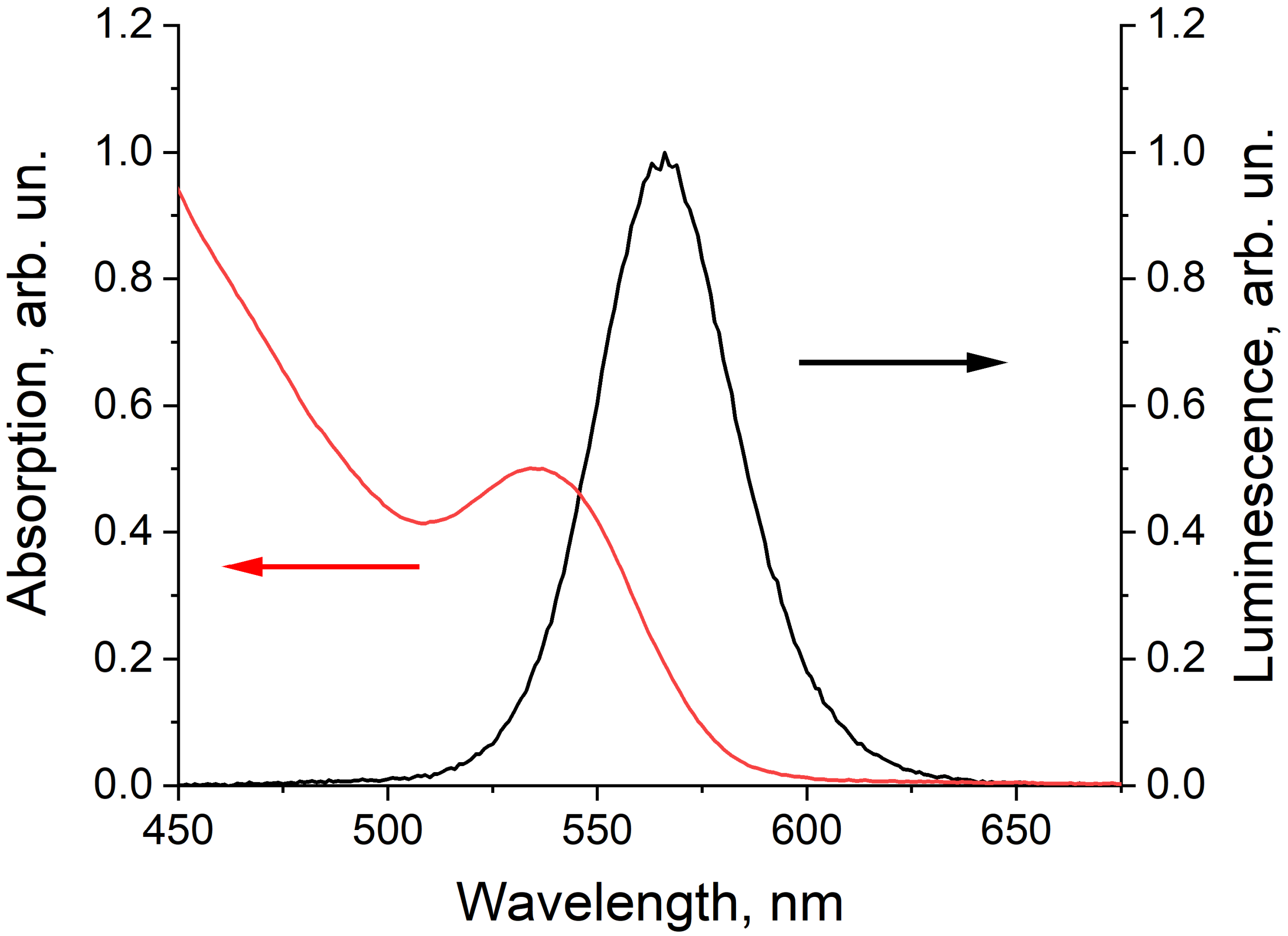

Figure S1. Luminescence (black curve) and absorption (red curve) spectra of the synthesized CdSe/ZnS/CdS/ZnS QDs.

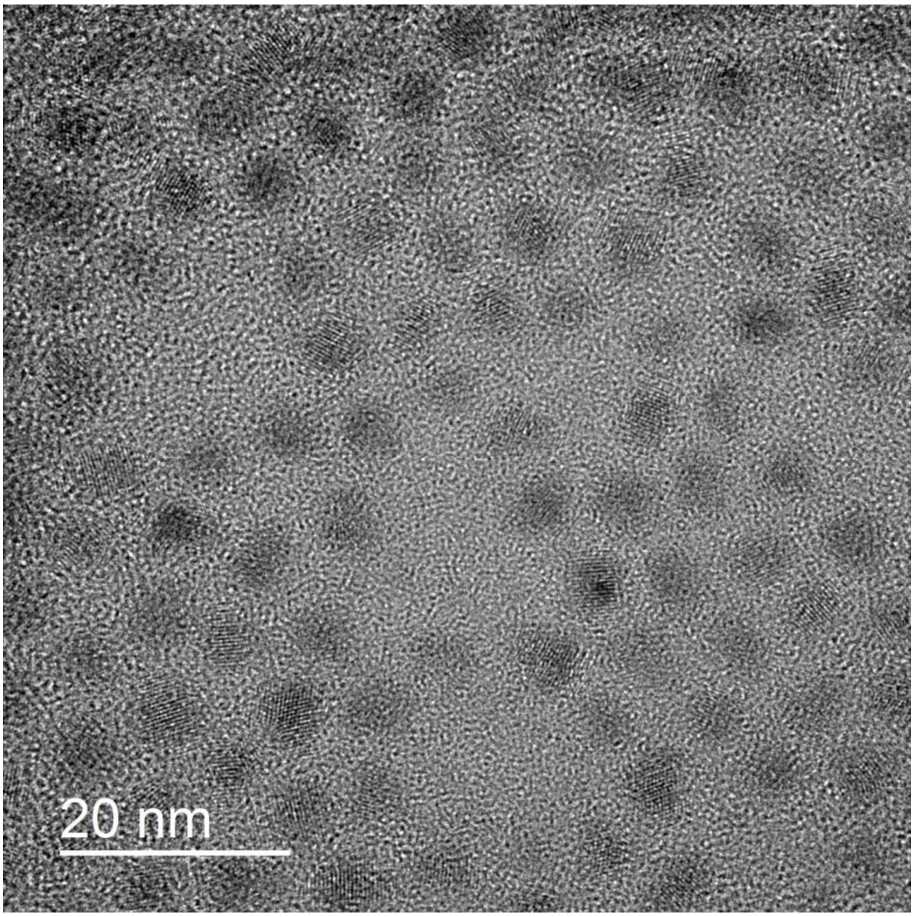

Figure S2. TEM image of the synthesized CdSe/ZnS/CdS/ZnS QDs.

Estimation of the thickness of PMMA electron blocking layers

In our previous work by Krivenkov et al. [1] we have performed a series of AFM measurements of the thicknesses of PMMA layers deposited from solutions in toluene by spin-coating onto glass substrates at fixed spinning rate of 3500 RPM. In this work we cannot directly use those data since we used deposition conditions different from those applied in previous work, namely the types of solvent (acetone) and substrate (unfinished QDLED structure with active QD layer exposed). Yet, those studies clearly demonstrate that the thickness of PMMA films deposited by spin-coating from PMMA solutions having different concentration shows a linear dependence on the latter in a wide concentration range. In [2], Dai et al. have deposited PMMA EBL from acetone solution with a concentration of 1.8 mg/mL. The thickness of this EBL indirectly obtained using FTIR absorbance values at ~1725 cm^{-1}, corresponding to the stretching vibrations of carbonyl groups, from a reference 90 nm PMMA layer on CaF_2 substrate and the one measured from PVK/QDs/PMMA stack mimicking the real device, was determined to be 6 nm. Therefore, assuming the linear dependence of film thickness on the concentration of deposition solution at constant spinning rate, and zero film thickness at "zero" polymer concentration, we can propose the following equation (1) for the thickness of PMMA layers (*D*) deposited from acetone solution with a concentration (*C*):

$$D = D_0/C_0 \times C \quad (1)$$

where D_0 is the reference film thickness, measured from solution with a known concentration C_0. By substitution of the reference data from Dai et al. (6 nm and 1.8 mg/mL for D_0 and C_0, respectively) into (1), we have obtained equation (2) for the thickness of PMMA layers deposited from acetone at 2000 RPM:

$$D = 3.33 \times C \quad (2)$$

Taking into account the approximate inverse square root dependence of the thickness of thin films deposited by spin coating on the substrate spinning rate [3], we can transform equation (2) into (3), which is suitable for estimation of PMMA layer thickness deposited from acetone solutions at 3500 RPM, by introducing a multiplier of 0.76, equal to the inverse square root of the spinning rates ratio (3500:2000):

$$D = 2.52 \times C \quad (3)$$

By substitution of PMMA concentrations used in this work, we can estimate the thicknesses of PMMA EBL in our devices, as shown in Table S1.

Table S1. Estimated PMMA EBL layer thickness deposited from PMMA solutions in acetone with different concentrations.

**PMMA solution concentration, mg/mL**	1.2	0.8	0.4	0.2	0.1	0.05
**PMMA EBL thickness, nm**	3.02	2.02	1.01	0.5	0.25	0.13

References

1. Krivenkov, V.; Goncharov, S.; Samokhvalov, P.; Sánchez-Iglesias, A.; Grzelczak, M.; Nabiev, I.; Rakovich, Y. Enhancement of Biexciton Emission Due to Long-Range Interaction of Single Quantum Dots and Gold Nanorods in a Thin-Film Hybrid Nanostructure. *J. Phys. Chem. Lett.* **2019**, *10*, 481–486, doi:10.1021/acs.jpclett.8b03549.
2. Dai, X.L.; Zhang, Z.X.; Jin, Y.Z.; Niu, Y.; Cao, H.J.; Liang, X.Y.; Chen, L.W.; Wang, J.P.; Peng, X.G. Solution-processed, high-performance light-emitting diodes based on quantum dots. *Nature* **2014**, *515*, 96-+, doi:10.1038/nature13829.
3. Emslie, A.G.; Bonner, F.T.; Peck, L.G. Flow of a Viscous Liquid on a Rotating Disk. *J. Appl. Phys.* **1958**, *29*, 858–862, doi:10.1063/1.1723300.
